# Successful tumour necrosis factor (TNF) blocking therapy suppresses oxidative stress and hypoxia-induced mitochondrial mutagenesis in inflammatory arthritis

**DOI:** 10.1186/ar3424

**Published:** 2011-07-25

**Authors:** Monika Biniecka, Aisling Kennedy, Chin T Ng, Ting C Chang, Emese Balogh, Edward Fox, Douglas J Veale, Ursula Fearon, Jacintha N O'Sullivan

**Affiliations:** 1 Translation Rheumatology Research Group, Dublin Academic Medical Centre, The Conway Institute of Biomolecular and Biomedical Research, St. Vincent's University Hospital, Elm Park, Dublin 4, Ireland; 2 Department of Pathology, University of Washington, 1959 NE Pacific St, HSB k056, Seattle, WA 98195, USA; 3 Department of Surgery, Institute of Molecular Medicine, Trinity Centre for Health Sciences, St James's Hospital, St James's Hospital, St James's Street, Dublin 8, Ireland

## Abstract

**Introduction:**

To examine the effects of tumour necrosis factor (TNF) blocking therapy on the levels of early mitochondrial genome alterations and oxidative stress.

**Methods:**

Eighteen inflammatory arthritis patients underwent synovial tissue oxygen (tpO_2_) measurements and clinical assessment of disease activity (DAS28-CRP) at baseline (T0) and three months (T3) after starting biologic therapy. Synovial tissue lipid peroxidation (4-HNE), T and B cell specific markers and synovial vascular endothelial growth factor (VEGF) were quantified by immunohistochemistry. Synovial levels of random mitochondrial DNA (mtDNA) mutations were assessed using Random Mutation Capture (RMC) assay.

**Results:**

4-HNE levels pre/post anti TNF-α therapy were inversely correlated with *in vivo *tpO_2 _(*P *< 0.008; r = -0.60). Biologic therapy responders showed a significantly reduced 4-HNE expression (*P *< 0.05). High 4-HNE expression correlated with high DAS28-CRP (*P *= 0.02; r = 0.53), tender joint count for 28 joints (TJC-28) (*P *= 0.03; r = 0.49), swollen joint count for 28 joints (SJC-28) (*P *= 0.03; r = 0.50) and visual analogue scale (VAS) (*P *= 0.04; r = 0.48). Strong positive association was found between the number of 4-HNE positive cells and CD4+ cells (*P *= 0.04; r = 0.60), CD8+ cells (*P *= 0.001; r = 0.70), CD20+ cells (*P *= 0.04; r = 0.68), CD68+ cells (*P *= 0.04; r = 0.47) and synovial VEGF expression (*P *= 0.01; r = 063). In patients whose *in vivo *tpO_2 _levels improved post treatment, significant reduction in mtDNA mutations and DAS28-CRP was observed (*P *< 0.05). In contrast in those patients whose tpO_2 _levels remained the same or reduced at T3, no significant changes for mtDNA mutations and DAS28-CRP were found.

**Conclusions:**

High levels of synovial oxidative stress and mitochondrial mutation burden are strongly associated with low *in vivo *oxygen tension and synovial inflammation. Furthermore these significant mitochondrial genome alterations are rescued following successful anti TNF-α treatment.

## Introduction

Mitochondria produce ATP through oxidative metabolism to provide cells with energy under physiological conditions. The mitochondrial electron transport chain (ETC) is also a major cellular source of reactive oxygen species (ROS) as some of the electrons passing to molecular oxygen are prone to leakage from the chain and get trapped by oxygen, which converts to superoxide [1]. Hypoxia characterised by an inadequate supply of molecular oxygen, can trigger mitochondria dysfunction through ineffective functioning of respiratory complexes of ETC [2,3].

Free oxygen radicals are highly active molecules and increased mitochondrial ROS generation promotes cellular oxidative stress resulting in oxidative mitochondrial DNA (mtDNA) damage and lipid peroxidation. Moreover, ROS mediate the stress signalling pathways involving nuclear factor-kappa B (NF-κB) [4]. mtDNA is in the proximity of ROS generation site and has relatively limited repair capacity, which makes it vulnerable to high mutation rates [5]. Mutations and deletions of the mitochondrial genome in genes encoding proteins for subunits of mitochondrial respiratory chain complexes I-V, rRNA and tRNA have been linked to a variety of degenerative human diseases and high levels of mtDNA mutations have been also found in many tumours and cancer cells [5,6].

Oxidative stress, which arises from an imbalance between ROS production and antioxidant defences, results also in lipid peroxidation of cell membrane polyunsaturated fatty acids [7]. The primary products of free-radical attack of biological membranes are lipid hydroperoxides, which can decompose to highly reactive, cytotoxic secondary end products, such as 4-hydroxy-2-nonenal (4-HNE) [8]. 4-HNE is an endogenously generated α,β unsaturated aldehyde, which is not only a marker of extensive oxidative stress but also can modulate cellular metabolism, inflammatory responses and apoptosis via its effects on transcriptional regulation and protein modification [9]. 4-HNE-induced mitochondrial protein modifications include those involved in the ETC, cellular respiration and Krebs cycle [10]. Moreover, 4-HNE can form adducts on DNA bases and modifies mtDNA thus measurement of such modifications may reflect the level of mitochondrial alterations [11].

Inflammatory arthritis (IA) is a chronic, progressive disorder associated with joint inflammation, synovial tissue hypertrophy, joint effusions and degradation of articular cartilage and bone. The normal synovial tissue is a relatively acellular structure with a lining layer (one to two cells thick) comprised of macrophages and fibroblasts. The morphology of IA synovium is strikingly different. There is a significant increase in the number of blood vessels that are associated with differential vascular morphology. Furthermore, the early vascular changes are accompanied by increased recruitment of macrophages and synovial fibroblast cells in the lining layer, along with infiltration of T, B and plasma cells. The precise mechanisms involved in regulation of persistent synovial infiltration and invasion are unclear, but high levels of TNF-α may be crucial in mediating the pathogenesis of IA. TNF-α is a proinflammatory cytokine, activating the NF-κB pathway, leading to a downstream cascade of other proinflammatory cytokines [12,13]. Moreover, it is known to increase mitochondrial ROS production [14,15] and induce the formation of lipid-derived aldehydes [16]; however TNF-α-induced mitochondrial mutagenesis has not yet been examined in patients with IA. Current targeted biologic therapies, including anti-TNF-α inhibitors result in greater disease improvement and prevention of joint erosion, although clinical studies on the efficacy of TNF-α blocking agents clearly show that about 40% of patients receiving this therapy are non-responders.

Recently, we demonstrated that successful biologic therapy significantly improves *in vivo *synovial hypoxia and it is strongly associated with improvement of joint inflammation [17]. In this study we investigate if successful anti-TNF-α treatment alters the levels of early mitochondrial genome alterations, which can play a crucial role in governing clinical response or resistance. Furthermore, we determine if TNF-α blocking therapy changes the levels of synovial 4-HNE, further confirming the relation between hypoxia, oxidative damage and mitochondrial mutagenesis.

## Materials and methods

### Patient recruitment

All research was carried out in accordance with the Declaration of Helsinki, and approval for this study was granted by the St. Vincent's University Hospital Medical Research and Ethics Committee. Eighteen patients with active IA (rheumatoid arthritis (RA) *n *= 14 and psoriatic arthritis (PsA) *n *= 4) were recruited from outpatient clinics at Department of Rheumatology, St. Vincent's University Hospital. All patients fulfilled the diagnostic criteria for RA and PsA [18,19]. All patients provided fully informed consent and underwent arthroscopy at baseline (T0) and three months after commencement of TNF blocking therapy (T3). At baseline, 50% of patients were naive for disease-modifying anti-rheumatic drugs (DMARDs) and corticosteroids; however, all patients including those on DMARDs (methotrexate (MTX) alone 35%, MTX + salazopyrine 10%, and plaquenil alone 5%) were biologic naive, had active disease, had at least one inflamed knee joint and were due to commence biologic therapy. Clinical and laboratory assessment was performed using standard measures of 28 tender and swollen joint count (DAS28), rheumatoid factor, anti-cyclic citrullinated peptide antibody, erythrocyte sedimentation rate (ESR), C-reactive protein (CRP) and global health visual analogue scale (VAS). All measurements were obtained on the same day prior to baseline and three months after anti TNF-α treatment arthroscopy.

### Arthroscopy, measurement of *in vivo *tpO_2 _and sample collection

Under local anaesthetic, patients (*n *= 18) underwent arthroscopy at baseline and three months after commencement of TNF blocking therapy. Arthroscopy of the inflamed knee was performed using a Wolf 2.7 mm needle arthroscope. Macroscopic synovitis and vascularity were scored on a VAS (0-100 mm). A LICOX^® ^combined pO_2 _and temperature probe (Integra Life Sciences Corporation, New Jersey, USA) was used to obtain synovial tissue oxygen partial pressure as previously described [20]. Synovial membrane biopsies were obtained from the site of the oxygen tension measurement and immediately embedded in mounting media for immunohistochemical analysis or snap frozen in liquid nitrogen for mitochondrial mutagenesis analysis.

### Immunohistochemistry and scoring

Immunohistochemistry was performed using 7 μm cryostat synovial tissue sections and the DAKO ChemMate Envision Kit (DAKO, Glostrup, Denmark). Sections were defrosted at room temperature for 20 minutes, fixed in acetone for 10 minutes and washed in PBS for 5 minutes. Non-specific binding was blocked using 10% casein in PBS for 20 minutes. The sections were incubated with primary antibodies against human 4-HNE (Genox, Baltimore, MD, USA), CD4, CD8, CD20, CD68 (all from DAKO, Glostrup, Denmark) and vascular endothelial growth factor (VEGF) (Santa Cruz Biotechnology, Inc., Santa Cruz, CA, USA). IgG control antibodies were used as negative controls. Following primary antibody incubation endogenous peroxidase activity was blocked using 0.3% hydrogen peroxide for seven minutes at room temperature. Slides were incubated with secondary antibody/HRP (DAKO, Glostrup, Denmark). DAB (1:50) was used to visualise staining, and Mayer's haematoxylin (BDH Laboratories, Poole, UK) was incubated for 30 seconds as a counterstain prior to mounting in Pertex mounting media. Images were captured using Olympus DP50 light microscope and AnalySIS software (Soft Imaging System Corporation, Lakewood, CO, USA). Slides were scored separately for lining and sublining layers using well established and validated semi-quantitative scoring method, where the percentage of cells that were positive for a specific marker was compared with the percentage of cells that were negative [21]. Percentage positivity was graded using 0 to 4 scale, where 0 represented no stained cells, 1 was 1 to 25% stained cells, 2 was 25 to 50% stained cells, 3 was 50 to 75% stained cells, and 4 was 75 to 100% stained cells.

### Mitochondrial random mutation capture assay

A sub-group of eight patients were selected from the initial cohort to quantify the levels of mitochondrial point mutations before and after treatment. Levels of mitochondrial point mutations in snap frozen synovial biopsies were analysed in a blinded fashion using Mitochondrial Random Mutation Capture assay as described previously [22]. Biopsies were homogenised (Precellys 24, Stretton Scientific Ltd., Stretton, Derbyshire, United Kingdom) in 10 mM Tris-HCl, pH 8.0, 150 mM NaCl, 20 mM EDTA, 0.5% SDS buffer and digested with Proteinase K (Sigma-Aldrich, Dublin, Ireland) at a final concentration of 0.2 mg/ml and incubated overnight at 56°C. The mtDNA was extracted using phenol-chloroform-isoamyl alcohol (25:24:1 by volume, Sigma-Aldrich, Dublin, Ireland) added in a 1:1 ratio with the lysed tissue, mixed thoroughly by shaking, and centrifuged at more than 12,000 × *g *for 10 minutes. The aqueous phase was gently removed from the top of the solution, without disturbing the interphase. The aqueous solution was again mixed with phenol-chloroform-isoamyl alcohol in a 1:1 ratio and re-extracted. One-tenth volume of 3 M sodium acetate was added, and the samples were precipitated with 2 to 2.5 volumes of ethanol. The DNA samples were resuspended in 50 μl 10 mM TrisCl. Ten micrograms of mtDNA were digested with 100 units of TaqαI restriction enzyme (New England BioLabs, Herts, United Kingdom), 1 × BSA and a TaqαI-specific digestion buffer (10 mM Tris-HCl, 10 mM MgCl_2_, 100 mM NaCl, pH 8.4) for 10 hours; 100 units of TaqαI being added to the reaction mixture every hour.

PCR amplification was performed in 25 μl reactions, containing 12.5 μl 2 × SYBR Green Brilliant Mastermix (Stratagene, Agilent Technologies, Inc., Santa Clara, CA, USA), 0.1 μl UDG (New England Biosciences, Herts, United Kingdom), 0.7 μl of 10 pM/μl forward and reverse primers (Integrated DNA Technologies, Inc., San Diego, CA, USA), and 6.7 μl water. The samples were amplified using a Roche Lightcycler 480 using the following protocol: 37°C for 10 minutes and 95°C for 10 minutes followed by 45 cycles of 95°C for 15 seconds, 60°C for 1 minute. Samples were held at 72°C for 7 minutes and, following melt curve analysis, immediately stored at -80°C. The primer sequences used were as follows: for mtDNA copy number: 5'ACAGTTTATGTAGCTTACCTCC-3' (forward) and 5'-TTGCTGCGTGCTTGATGCTTGT-3' (reverse); for random mutations: 5'-CCTCAACAGTTAAATCAACAAAACTGC-3' (forward) and 5'-GCGCTTACTTTGTAGCCTTCA-3' (reverse).

### Statistical analysis

Data are presented as medians and interquartile ranges. Data were assessed using Wilcoxon's signed-rank test or Spearman's rank correlation coefficient as appropriate using the Statistical Package for the Social Sciences (SPSS, Chicago, IL, USA). All *P *values are two-sided and *P *values less than 0.05 were considered statistically significant.

## Results

### *In vivo *changes of oxidative stress pre/post anti TNF-α therapy

Eighteen IA patients underwent synovial tissue oxygen tension (tpO_2_) measurements and clinical assessment of disease activity (28-joint count disease activity score using C-reactive protein (DAS28-CRP)) at baseline and three months after starting biologic therapy. At T3 patients were categorised according to remission criteria using the DAS28 cut-off less than or more than 2.6. Patients with DAS28-CRP less than 2.6 were defined as responders (*n *= 7) and patients with DAS28-CRP more than 2.6 were defined as non-responders (*n *= 11). In responders, the median baseline pO_2 _in the synovial tissue was 18.07 mmHg (range 4.3 to 42.2 mmHg), and was lower than in those patients at T3 (median tpO_2 _39.25 mmHg (range 24.7 to 68.2 mmHg)). Of clinical responders, 86% had a corresponding increase in their synovial tpO_2 _measurements. In non-responders the median baseline pO_2 _was 23.75 mmHg (range 6.8 to 46.4 mmHg), and their median pO_2 _level after biologic therapy was 19.78 mmHg (range 10.5 to 39.6 mmHg). In clinical non-responders, 64% patients showed decrease in their synovial tpO_2 _levels at T3. Furthermore, tpO_2 _levels did not differ significantly between baseline patients with RA and those with PsA (*n *= 14 RA, *n *= 4 PsA). The median oxygen tension for RA was 23.5 mmHg and for PsA was 14.5 mmHg (*P *= 0.3).

**Figure 1:**
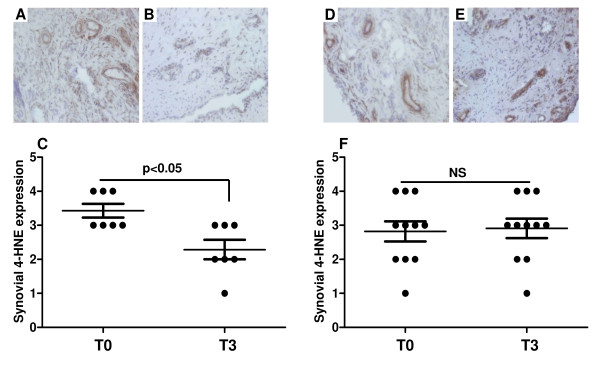
**Representative pre/post immunohistochemical images of 4-HNE expression and their graphical representation**. **(a to c) **Responders. **(d to f) **Non-responders. T0 is time at baseline; T3 is three months after anti-TNF-α treatment. **(a to b) **Biologic therapy responders showed lower synovial 4-hydroxy-2-nonenal (4-HNE) expression at **(b) **T3 compared with their **(a) **T0 levels. **(c) **Graphical illustration of synovial 4-HNE levels at T0 and T3 (*P *< 0.05). **(d to e) **No significant 4-HNE changes were seen between **(d) **T0 and **(e) **T3 in patients who did not respond to therapy. **(f) **Graphical representation of synovial 4-HNE levels in non-responders at T0 and T3.

To determine whether biologic treatment changes the levels of synovial oxidative damage, the number of 4-HNE positive cells was assessed in both lining and sublining layers of synovial tissue. Figures 1a and 1b show representative images of 4-HNE expression levels in responders at T0 and T3, respectively. Figure 1c graphically illustrates significantly reduced cytoplasmic 4-HNE expression in sublining layer in patients who successfully responded to anti-TNF-α therapy (*P *< 0.05). No significant differences in the levels of cytoplasmic 4-HNE expression pre/post therapy were found in non-responders (Figures 1d to 1f). In addition, the levels of 4-HNE did not differ significantly between baseline patients with RA and those with PsA (*P *= 0.6).

Previously, we demonstrated significant baseline inverse correlation between tpO_2 _measurements and 4-HNE expression [20]. In this study we extend these findings and demonstrate that change in tpO_2 _is also significantly and inversely correlated with changes in 4-HNE levels pre/post biologic therapy (*P *< 0.008; r = -0.60; Table 1). It suggests that as synovial tissue becomes less hypoxic oxidative stress is decreased. Furthermore, when patients were categorised according to their changes in tpO_2 _before and after therapy, a significant reduction in the number of 4-HNE positive cells was observed only in patients who had higher oxygen levels at T3 compared with T0 (data not shown).

**Table 1:** Spearman's rank test correlations of 4-HNE microscopic scores in synovial tissue pre/post anti TNF-α therapy with clinical parameters

4-HNE	r-value	*P *value
tpO_2_	-0.60	0.008
DAS28-CRP	0.53	0.02
TJC-28	0.49	0.03
SJC-28	0.50	0.03
VAS	0.48	0.04

DAS28-CRP, 28-joint count disease activity score using C-reactive protein; 4-HNE, 4-hydroxy-2-nonenal; SJC-28, swollen joint count for 28 joints; TJC-28, tender joint count for 28 joints; tpO_2_, *in vivo *tissue oxygen tension; VAS, visual analogue scale.

### Synovial oxidative stress and clinical markers

The relation of oxidative stress marker and clinical markers pre/post anti-TNF-α therapy is shown in Table 1. We found significant positive correlations between levels of 4-HNE and DAS28-CRP (*P *= 0.02; r = 0.53), 4-HNE and tender joint count (TJC)-28 (*P *= 0.03; r = 0.49), 4-HNE and swollen joint count (SJC)-28 (*P *= 0.03; r = 0.50), 4-HNE and VAS (*P *= 0.04; r = 0.48). These results demonstrate a link between oxidative stress and clinical parameters of disease activity and suggest that microscopically assessed levels of 4-HNE may closely reflect clinical scores of IA.

### Synovial levels of oxidative stress, inflammation and angiogenesis pre/post biologic therapy

Levels of lipid peroxidation were correlated with specific markers of T-cells (CD4 and CD8), B-cells (CD20), and macrophages (CD68). Table 2 demonstrates significant positive associations between the number of 4-HNE positive cells and CD4^+ ^cells (*P *= 0.04; r = 0.60), CD8^+ ^cells (*P *= 0.001; r = 0.70), CD20^+ ^cells (*P *= 0.04; r = 0.68) and CD68^+ ^cells (*P *= 0.04; r = 0.47). Furthermore, high 4-HNE expression correlates with high level of VEGF angiogenic marker (*P *= 0.01; r = 0.63; Table 2). We have also performed the colocalisation staining between synovial 4-HNE and all cellular specific markers and observed 4-HNE expression in T-cells, B-cells, macrophages and cells of blood vessels.

**Table 2:** Spearman's rank test correlations of 4-HNE synovial tissue pre/post anti TNF-α therapy with synovial inflammation and angiogenesis

4-HNE	r-value	*P *value
CD4 ll	0.60	0.04
CD8 sl	0.70	0.001
CD20 sl	0.68	0.04
CD68 ll	0.47	0.04
VEGF bv	0.63	0.01

bv, blood vessel; CD4 and CD8, T-cell markers; CD20, B-cell marker; CD68, marker of macrophages; 4-HNE, 4-hydroxy-2-nonenal; ll, lining layer; sl, sublining layer; VEGF, vascular endothelial growth factor.

As higher levels of 4-HNE are strongly associated with high VEGF expression and the number of inflammatory cells pre/post therapy, it may suggest a key role of oxidative stress in driving inflammation and angiogenesis, two crucial processes involved in progression of IA.

### Effect of biologic therapy on mitochondrial mutagenesis

**Figure 2:**
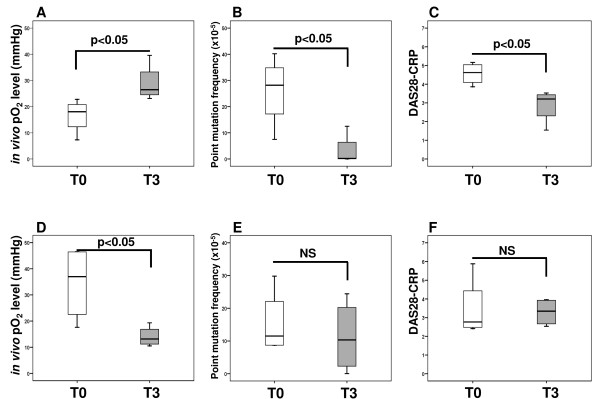
**Effects of anti TNF-α therapy on the levels of mitochondrial point mutation and disease activity (DAS28-CRP)**. Patients were categorised into two groups according to their *in vivo *tissue oxygen tension (tpO_2_) changes from baseline (T0 - white boxes) to three months after anti TNF-α therapy (T3 - grey boxes). **(a) **Group 1 represents patients whose tpO_2 _levels improved at T3 in comparison with T0 (*n *= 4; *P *< 0.05). **(b) **Increase in tpO_2 _was associated with significantly reduced frequency of mitochondrial point mutations at T3 in comparison with baseline levels (*P *< 0.05). **(c) **It was also associated with significantly lower DAS28-CRP scores at T3 than at T0 (*P *< 0.05). **(d) **Group 2 represents patients whose *in vivo *oxygen levels remained the same or reduced at T3 in comparison with T0 (*n *= 4; *P *< 0.05). **(e) **No significant changes in the pre/post levels of mtDNA mutations were observed in patients having more hypoxic synovium at T3 than at T0 (NS). **(f) **No significant changes in the pre/post levels of DAS28-CRP were found in patients who were more hypoxic at T3 than at T0 (NS). Boxes represent the 25th to 75th percentiles, lines within the boxes represent the median, and lines outside the boxes represent the 10th and 90th percentiles.

To determine whether biologic therapy alters mitochondrial genome instability, random mutation capture assay was performed at baseline and three months after treatment in a sub-group of eight patients. Patients were categorised into two groups, those whose tpO_2 _levels improved after treatment (*n *= 4) and those whose *in vivo *oxygen level remained the same or reduced after three months therapy (*n *= 4). Figure 2a shows pre/post tpO_2 _changes in patients who had a significant increase in *in vivo *oxygen measurements after treatment in comparison with their baseline levels (*P *< 0.05). This was associated with significantly reduced frequency of mitochondrial point mutations in comparison with baseline levels (*P *< 0.05; Figure 2b) and with significantly lower DAS28-CRP scores at T3 than before treatment (*P *< 0.05; Figure 2c). In contrast, no significant changes in the pre/post levels of mtDNA mutations (Figure 2e) and DAS28-CRP (Figure 2f) were observed in patients who showed no improvement in *in vivo *tpO_2 _levels post treatment (*P *< 0.05; Figure 2d). This data may suggest mitochondrial genome alterations as a consequence of elevated synovial hypoxia. In addition, we found that hypoxia-induced mitochondrial mutagenesis was positively correlated with clinical markers of IA. As shown in Table 3 we found significant associations between the levels of mitochondrial point mutations and DAS28-CRP (*P *= 0.01; r = 0.83), CRP (*P *= 0.02; r = 0.77) and ESR (*P *= 0.04; r = 0.73).

**Table 3:** Spearman's rank test correlations of mitochondrial point mutations pre/post anti TNF-α therapy with clinical parameters

Mitochondrial point mutations	r-value	*P *value
DAS28-CRP	0.83	0.01
CRP (mmg/L)	0.77	0.02
ESR (mm/hr)	0.73	0.04

CRP, C-reactive protein; DAS28-CRP, 28-joint count disease activity score using C-reactive protein; ESR, erythrocyte sedimentation rate.

## Discussion

Chronic inflammatory arthropathies, such as RA and PsA, are characterised by complex chronic inflammatory processes. Oxygen metabolism is important in synovitis and joint destruction [23]. ROS stimulates synovial fibroblasts to secrete matrix metalloproteinases, inhibits cartilage proteoglycan synthesis and accelerates bone resorption [24,25]. Previously, we have demonstrated profoundly hypoxic synovial environment of the inflamed joint (approximately 3%) [26]. Furthermore, we have shown that biologic anti-TNF-α therapy significantly increased the synovial *in vivo *tpO_2 _levels only in those patients who respond to anti-TNF-α therapy [17]. In this study we examine the effect of TNF-blocking therapy on mitochondrial mutagenesis and synovial oxidative stress profiles. We report for the first time that the increase in tpO_2 _levels observed in responders is associated with significant decrease and strong inverse correlation of synovial lipid peroxidation. In addition, increases in tpO_2 _significantly reduces the levels of random mitochondrial mutations, presumably as a result of decreased oxidative stress profile.

TNF-α affects many cellular processes, such as activation of phospholipases [27], proteases [28] and DNA damage [29]. Mitochondrially derived ROS are strongly implicated in TNF-α cytotoxicity and may mediate the activation of transcriptional factor NF-κB, which in turn can stimulate mitochondrial NADPH oxidase [15,30]. Inhibition of ETC complex III by antimycin A increases ROS and inhibits TNF-α triggered NF-κB activation, highlighting the importance of the ETC in TNF-α cytotoxicity [31]. Recently, we have shown that hypoxia is an important stimulus of TNF-α secretion, where higher levels of synovial fluid TNF-α were detected in patients with synovial tpO_2 _less than 20 mmHg than in those with tpO_2 _more than 20 mmHg [26].

Oxidative stress arising from overproduction of ROS leads to formation of reactive aldehydes such as 4-HNE. Mitochondrial are primed for attack by 4-HNE and formation of adducts between 4-HNE and mitochondrial components. Detection of 4-HNE-mitochondrial protein adducts can reflect mitochondrial dysfunction and oxidative stress [32]. We have previously assessed the expression of synovial lipid peroxidation in IA patients and demonstrated a significant inverse correlation between 4-HNE expression and oxygen tension of the inflamed join, probably reflecting mitochondrial damage [20]. Mitochondrial membrane components are targets for 4-HNE modification and the adenine nucleotide translocator in the inner mitochondrial membrane is affected by lipid peroxidation [33]. This study in the first to show that patients who respond to TNF-blocking therapy show a significant increase in tpO_2 _and this is associated with reduced 4-HNE levels. In contrast, in non-responders there is no change in *in vivo *oxygen levels and subsequently no change in 4-HNE levels. These data suggest that as the joint tissue becomes less hypoxic, a corresponding reduction in oxidative stress is affected. Previous studies have demonstrated positive effects of anti-TNF-α treatment on oxidative damage in RA, where urinary levels of oxidative DNA damage and lipid peroxidation were significantly reduced at three months therapy [34]. However, our study considerably extends the above reports and shows direct evidence of a significant reduction of oxidative stress in relation to *in vivo *hypoxia measurements.

We have recently demonstrated that increased tpO_2 _levels after successful anti-TNF biologic therapy is associated with reduced disease activity and macroscopic vascularity [17]. Furthermore, we have also reported that high synovial 4-HNE levels positively correlated with clinical disease activity scores in patients prior to receiving TNF-α blocking therapy [20]. In this study the same parameters were assessed in patients after anti-TNF-α treatment and we found significant positive association between synovial 4-HNE expression and clinical measures of arthritis.

Several cellular and environmental sources of synovial oxidative stress have been proposed, including activated neutrophils, monocytes and macrophages, hypoxia and vascular changes. Furthermore, studies by Remans et al. indicated synovial T lymphocytes as the main generators of intracellular free radicals in RA patients [35]. We demonstrate a correlation between oxidative stress, inflammation and angiogenesis, where increase in tpO_2 _and reduce oxidative stress observed in responders is associated with lower microscopic scores of T-cells (CD4 and CD8), B-cells (CD20), macrophages (CD68) and angiogenesis (VEGF). Experiments using 4-HNE-modified antigens of T and B cells showed rapid autoimmune response, suggesting that B and T cell modification by 4-HNE may result in the onset of autoimmune reactions or even autoimmune disease processes [36]. The link between oxidative lipid modifications and activation of the inflammatory potential of macrophages has been also suggested [37]. In human osteoarthritic chondrocytes 4-HNE induces prostaglandin E release and cyclooxygenase-2 (COX-2) expression, providing evidence for the role of 4-HNE as redox-sensitive signalling mechanisms of inflammatory response [38]. Furthermore, 4-HNE elevated VEGF secretion has been shown in retinal pigment epithelial cells [39] and vascular smooth muscle cells [40]. This correlation of VEGF expression and 4-HNE supports our current findings.

RA has many features of autoimmune disease; however, some studies suggest inflammation-independent joint destruction [41]. It has been shown that elevated production of ROS at the sites of chronic inflammation has genotoxic effects and increases the likelihood of mutagenic events. In RA, local exposure to oxidative stress was found to induce genetic changes and was proposed as a mechanism that permanently alters and imprints synovial cells [42,43]. Furthermore, oxidative stress can suppress expression of DNA repair enzymes in inflamed synovium such as DNA mismatch repair system that might potentially limit the accumulation of mutations [44]. Other extensive studies demonstrated synovial *p53 *mutations, which are characteristic DNA damage caused by oxidative stress. High expression of *p53 *was found in synovial tissue from longstanding RA patients and lower in early RA patients, osteoarthritis (OA) and reactive arthritis patients [45]. This oxidative DNA damage of *p53 *gene is likely to promote neoplastic transformation of synovial cells that may subsequently contribute to disease progression and joint destruction.

Oxidative stress may also contribute to somatic mtDNA mutation. mtDNA mutations were known to have a key role in ageing-related diseases and carcinogenesis. Currently, there is a growing body of evidence suggesting the role of mitochondrial alterations in rheumatoid disorders [46]. Recent studies showed higher accumulation of mtDNA damage in chondrocytes from OA patients compared with those from normal donors [47]. Higher incidence of mtDNA somatic mutations has also been detected in synoviocytes and synovial tissue of RA than OA controls [48]; however, the frequency of mitochondrial mutations has not been examined. Recently, using synovial tissue of baseline IA patients, we have screened a large number of mtDNA molecules for the presence of unexpanded random mutations, which may be crucial in driving disease progression. We demonstrated, for the first time that greater levels of mtDNA point mutations were significantly associated with higher hypoxia *in vivo*, oxidative stress and disease activity [49].

TNF-α was demonstrated to induce *in vitro *mitochondrial ROS release and DNA damage in human chondrocytes and overexpression of the DNA repair enzyme prevents mtDNA alterations following TNF-α exposure [50]. In this study, we determined whether TNF therapy affect the levels of mtDNA mutations. We observed that the increase in tpO_2 _after treatment was associated with significant decrease in the levels of mtDNA mutations and reduction of disease activity scores DAS28-CRP. Contrary, no significant improvements in the levels of mtDNA mutations and DAS28-CRP were found in patients who had more hypoxic synovium after receiving TNF blocking treatment. Our findings strongly support the hypothesis that an increase in mutation frequency is a consequence of elevated hypoxia and oxidative damage to the mitochondrial genome. Furthermore, our results are in agreement with another report indicating the role of oxidative stress and diminished mtDNA integrity in the progression of OA, where high levels of mutagenesis following exposure to ROS were associated with reduced mtDNA capacity and cell viability [47]. In addition, our study is the first to show that successful anti-TNF-α therapy reduces the frequency of mitochondrial synovial mutagenesis in IA. It may suggest a central role of mitochondrial mutagenesis in the cellular mechanism of anti-TNF-α response or resistance to the treatment

## Conclusions

We have clearly demonstrated a close association between oxidative stress, mitochondrial mutagenesis and clinical responses to TNF-blocking therapy in IA patients. The greater mitochondrial mutation burden in synovial tissue is associated with higher hypoxia levels *in vivo *and these significant mitochondrial genome alterations are rescued following successful anti-TNF treatment.

## Abbreviations

4-HNE: 4-hydroxy-2-nonenal; CRP: C-reactive protein; DAS28-CRP: 28-joint count disease activity score using C-reactive protein; DMARDs: disease-modifying anti-rheumatic drug; ESR: erythrocyte sedimentation rate; ETC: electron transport chain; IA: inflammatory arthritis; mtDNA: mitochondrial DNA; MTX: methotrexate; NF-κB: nuclear factor-kappa B; OA: osteoarthritis; PBS: phosphate-buffered saline; PsA: psoriatic arthritis; RA: rheumatoid arthritis; ROS: reactive oxygen species; SJC-28: swollen joint count for 28 joints; T0: timepoint 0 or baseline; T3: timepoint three months after starting therapy; TJC-28: tender joint count for 28 joints; TNF-α: tumour necrosis factor alpha; tpO_2_: tissue oxygen partial pressure; VAS: visual analogue scale; VEGF: vascular endothelial growth factor.

## Competing interests

The authors declare that they have no competing interests.

## Authors' contributions

MB conducted most of the experiments and analysis of data. AK, CTN, TCC, EB, EF and UF performed some of the experiments. JNO, UF, DV and MB participated in the data analysis and manuscript preparation and final approval of the version to be published. JNO, UF and DV participated in the study design and supervised the research. DV and CTN recruited all patients, performed the arthroscopies and oxygen measurements and provided all clinical information. All authors read and approved the final manuscript.
